# Effect of extracorporeal shockwave therapy for rotator cuff tendinopathy: a systematic review and meta-analysis

**DOI:** 10.1186/s12891-024-07445-7

**Published:** 2024-05-04

**Authors:** Xiali Xue, Qingfa Song, Xinwei Yang, Amila Kuati, Hao Fu, Yulei Liu, Guoqing Cui

**Affiliations:** 1 School of Sports Medicine and Health, Chengdu Sport University, Chengdu, China; 2 Department of Sports Medicine, Peking University Third Hospital, Institute of Sports Medicine of Peking University, Beijing, China; 3 Chengdu University of Traditional Chinese Medicine, Chengdu, China; 4 Department of Rehabilitation, Peking University Third Hospital, Beijing, 100191 China

**Keywords:** Extracorporeal shock wave therapy, Rotator cuff tendinopathy, Shoulder, Rehabilitation, ESWT, Meta-analysis

## Abstract

**Background:**

Rotator cuff tendinopathy (RCT) is a widespread musculoskeletal disorder and a primary cause of shoulder pain and limited function. The resulting pain and limited functionality have a detrimental impact on the overall quality of life. The purpose of this study was to perform a systematic review of the effects of extracorporeal shock wave therapy (ESWT) for RCT.

**Methods:**

The literature search was conducted on the following databases from inception to February 20, 2024: PubMed, Web of Science, the Cochrane Library, Scopus, MEDLINE, EMBASE, EBSCO, and China National Knowledge Infrastructure (CNKI) were checked to identify the potential studies exploring the effect of ESWT for the treatment of Rotator cuff tendinopathy (Calcification or non-calcification), control group for sham, other treatments (including placebo), without restriction of date, language. Two researchers independently screened literature, extracted data, evaluated the risk of bias in the included studies, and performed meta-analysis using RevMan 5.3 software.

**Results:**

A total of 16 RCTs with 1093 patients were included. The results showed that compared with the control group, ESWT for pain score Visual Analogue Scale/Score (VAS) (SMD = -1.95, 95% CI -2.47, -1.41, *P* < 0.00001), function score Constant-Murley score (CMS) (SMD = 1.30, 95% CI 0.67, 1.92, *P* < 0.00001), University of California Los Angeles score (UCLA) (SMD = 2.69, 95% CI 1.64, 3.74, *P* < 0.00001), American Shoulder and Elbow Surgeons form (ASES) (SMD = 1.29, 95% CI 0.93, 1.65, *P* < 0.00001), Range of motion (ROM) External rotation (SMD = 1.00, 95% CI 0.29, 1.72, *P* = 0.02), Total effective rate (TER) (OR = 3.64, 95% CI 1.85, 7.14, *P* = 0.0002), the differences in the above results were statistically significant. But ROM-Abduction (SMD = 0.72, 95% CI -0.22, 1.66, *P* = 0.13), the difference was not statistically significant.

**Conclusion:**

Currently limited evidence suggests that, compared with the control group, ESWT can provide better pain relief, functional recovery, and maintenance of function in patients with RCT.

**Supplementary Information:**

The online version contains supplementary material available at 10.1186/s12891-024-07445-7.

## Introduction

Rotator cuff tendinopathy (RCT) is a common shoulder condition and one of the primary causes of shoulder pain and functional impairment [1]. The incidence of RCT in individuals aged 60 and above is approximately 20% to 50%, manifesting primarily as pain, limitations in daily activities, and reduced shoulder joint function [2, 3]. The etiology of RCT is multifactorial, and its pathogenesis is not fully understood. Common factors such as aging, overuse, mechanical shock, smoking, and family inheritance, and studies of familial susceptibility have shown that genetics also play a role in the pathogenesis of rotator cuff disease [4]. Injury and degeneration are two common mechanisms of RCT. Most chronic shoulder pain is caused by repeated impingement of the rotator cuff at the acromion. The early manifestations are local edema of the rotator cuff Hemorrhage, which then develops into tendinitis with localized fibrosis [5, 6]. If the influencing factors persist for a long time, it will eventually lead to a tear of the rotator cuff [7]. Therefore, effective treatment of RCT is crucial for restoring shoulder function, alleviating pain, and enhancing patient's quality of life. The treatment of RCT is mainly divided into surgical treatment and non-surgical treatment [8]. Available evidence suggests that both physical therapy and surgery can significantly improve patient-reported outcomes in symptomatic patients with small-to-moderate full-thickness RCT [6]. At present, great progress has been made in the non-surgical treatment of RCT. Non-surgical treatment mainly includes (1) physical therapy; (2) subacromial closed injection; (3) non-steroidal anti-inflammatory drugs; and (4) Traditional Chinese medicine preparations and acupuncture [9]. However, none of the treatments is simple, effective and non-invasive.

Extracorporeal shock wave therapy (ESWT) has been widely used as a treatment method for musculoskeletal tendon disorders [10]. Biological effects of ESWT have been reported to include tissue regeneration, wound healing, angiogenesis, bone remodeling, and anti-inflammation [11]. Its mechanism is similar to the cascade process triggered by mechanotransduction: mechanical energy causes changes in the cytoskeleton, causing a response in the nucleus (such as the release of mRNA), thereby affecting various cellular structures such as mitochondria, endoplasmic reticulum, and intracellular vesicles, enzymes Nootropic responses lead to improvements in the healing process [12]. Through ESWT coagulation, the adhesive tissue can be loosened to promote the rapid recovery of skeletal muscle injury and internal inflammation, so the analgesic effect of ESWT is more obvious. In recent years, shock waves have achieved remarkable results in the treatment of RCT, with the characteristics of non-invasiveness and high safety. ESWT is widely used in the field of rehabilitation, especially for improving chronic pain and tendinosis. It has a good therapeutic effect [13]. Indeed, ESWT emerges as a viable option for the treatment of RCT.

At present, the efficacy of ESWT in the treatment of RCT is still controversial. Some studies have indicated that extracorporeal shock waves have a significant effect in reducing pain, improving function and promoting tissue repair in patients with RCT [14, 15], while other studies have reached the opposite conclusion [16, 17]. Danilo et al.'s meta-analysis [18] found that, in short-term follow-up, ESWT showed a slight improvement in shoulder pain compared to sham ESWT. ESWT was not superior to sham ESWT in improving functionality, and it was also not superior to other treatments in improving both shoulder pain and function. There is still controversy regarding the effectiveness of ESWT, as there are few systematic reviews on the impact of extracorporeal shock waves on shoulder pain and function in patients with RCT, and the latest published studies are yet to be included. This study aims to systematically review and meta-analyze the effect of ESWT on shoulder pain and functional recovery in patients with RCT. The effectiveness of clinical efficacy and its scientific basis, hoping to provide a reference for future research in this field based on the research results.

## Methods

### Study protocol

This systematic review was performed following Preferred Reporting Items for Systematic Reviews and Meta-Analyses 2020 guidelines (PRISMA 2020) [19] (see Supplementary Material 1) and has been registered at PROSPERO (Identification number: CRD42023441407).

### Eligibility criteria

The inclusion criteria for this study encompassed all Randomized Controlled Trials (RCTs) that assessed the efficacy of ESWT in the treatment of RCT.

(1) Adult patients (18 years of age and older) with RCT will be included, consistent with clinical or radiographic findings, regardless of race, nationality, or course of disease. (2) RCTs comparing the effect of ESWT and other treatments (including placebo) for RCT. The experimental group was treated with ESWT and the control group was treated with a placebo ESWT or other treatments. (3) Outcome indicator: The main outcome indicator is the Visual Analogue Scale/Score (VAS), Secondary outcome indicators are the Constant-Murley score (CMS), University of California Los Angeles score (UCLA), Range of motion (ROM), American Shoulder and Elbow Surgeons form (ASES) and Total effective rate (TER). (4) Without restriction of date and language.

### Exclusion criteria

(1) Non-randomized control trials; (2) Animal experiments; (3) Incomparability between the intervention and control groups; (4) Letters, reviews, case reports, conference abstracts and comments. (5) if individuals who had a history of trauma or other conditions (partial or full rotator cuff tears, osteoarthritis, and adhesive capsulitis), systemic inflammation, or associated neurological diseases.

### Search strategy

The literature search was conducted on the following databases from inception to February 20, 2024: PubMed, Web of Science, the Cochrane Library, Scopus, MEDLINE, EMBASE, EBSCO, and China National Knowledge Infrastructure (CNKI) were checked to identify the potential studies exploring the effect of ESWT for the treatment of RCT. The search strategy uses the combination of subject words and free words, Boolean operators (AND or OR), and the search strategies of different databases are slightly different. Search terms included “Rotator cuff tendinopathy”, “Cuff Tendinopathy, Rotator”, “Rotator Cuff Tendinitis”, “Extracorporeal shock wave therapy”, “ESWT”, “Physical therapy modalities”, “Physical therapy”, “Randomized controlled trial,” “Controlled clinical trial,” “Randomized,” and “Trial.” Take Pubmed as an example, the specific retrieval strategy is displayed in Supplementary Material 2. Besides, the reference lists of eligible studies and relevant reviews were searched in case of possible missing articles.

### Selection process

Two experienced researchers (XLX and QFS) independently screened and evaluated the title and abstract of each study according to the established criteria, excluded unqualified literature, and then read the remaining full text and screened based on the previous content. Full text, determining criteria for eligible research. In case of disagreement, decisions were made by discussion with the corresponding author (GQC).

### Data collection process

Two researchers (XWY and FH) independently conducted data extraction. They used a pre-designed data collection form to record information including first author details, publication date, publication year, country of origin, study design, sample size, basic patient characteristics, intervention details for treatment and control groups, and primary and secondary outcomes. In cases where the above-mentioned data were incomplete, attempts were made to contact the article authors for additional information. When data were not reported, authors were emailed three times with one week in between attempts to clarify the information. In trials in which SD was not reported, the study will be excluded directly. Any disagreements during data extraction were resolved through discussions with a third researcher (GQC).

### Study risk of bias assessment

Two researchers (XLX and QFS), independently evaluated the methodological quality of each reviewed study using the Cochrane Risk of Bias tools 2.0 (ROB 2.0) to assess the risk of bias in randomized trials. Any discrepancies between their assessments were resolved through discussion, or consultation with a third researcher (GQC) was sought if a consensus could not be reached. The methodological quality of the studies was assessed across several domains, including the randomization process, adherence to intended interventions, handling of missing outcome data, measurement of outcomes, and selection of reported results. Each of these domains was categorized as Low risk, High risk, or Some concerns according to the ROB 2.0 criteria.

### Statistical analysis

Review Manager software (RevMan 5.3) was used for data analysis. Continuous variables were diagnosed with the Standardized mean difference (SMD) and 95% Confidence interval (CI), and the Odds Ratio (OR) was used for pooled analysis of dichotomous variables. SMD were classified as small (< 0.40), medium (between 0.41 and 0.70), and large (> 0.70). Statistical heterogeneity between studies will be assessed using P and I2 values, with *P* < 0.1 and I2 > 50% showing high heterogeneity, using a random effects model. When heterogeneity is not significant, a fixed effects model was used. If heterogeneity is high, subgroup analysis or meta-regression was performed to explore sources of heterogeneity. Funnel plots were applied for the assessment of publication bias. The extracted data was input into the computer, reviewed, and independently analyzed by two researchers. The meta-analysis is set at *P* < 0.05 for the significance level.

## Results

### Study selection

**Fig. 1:**
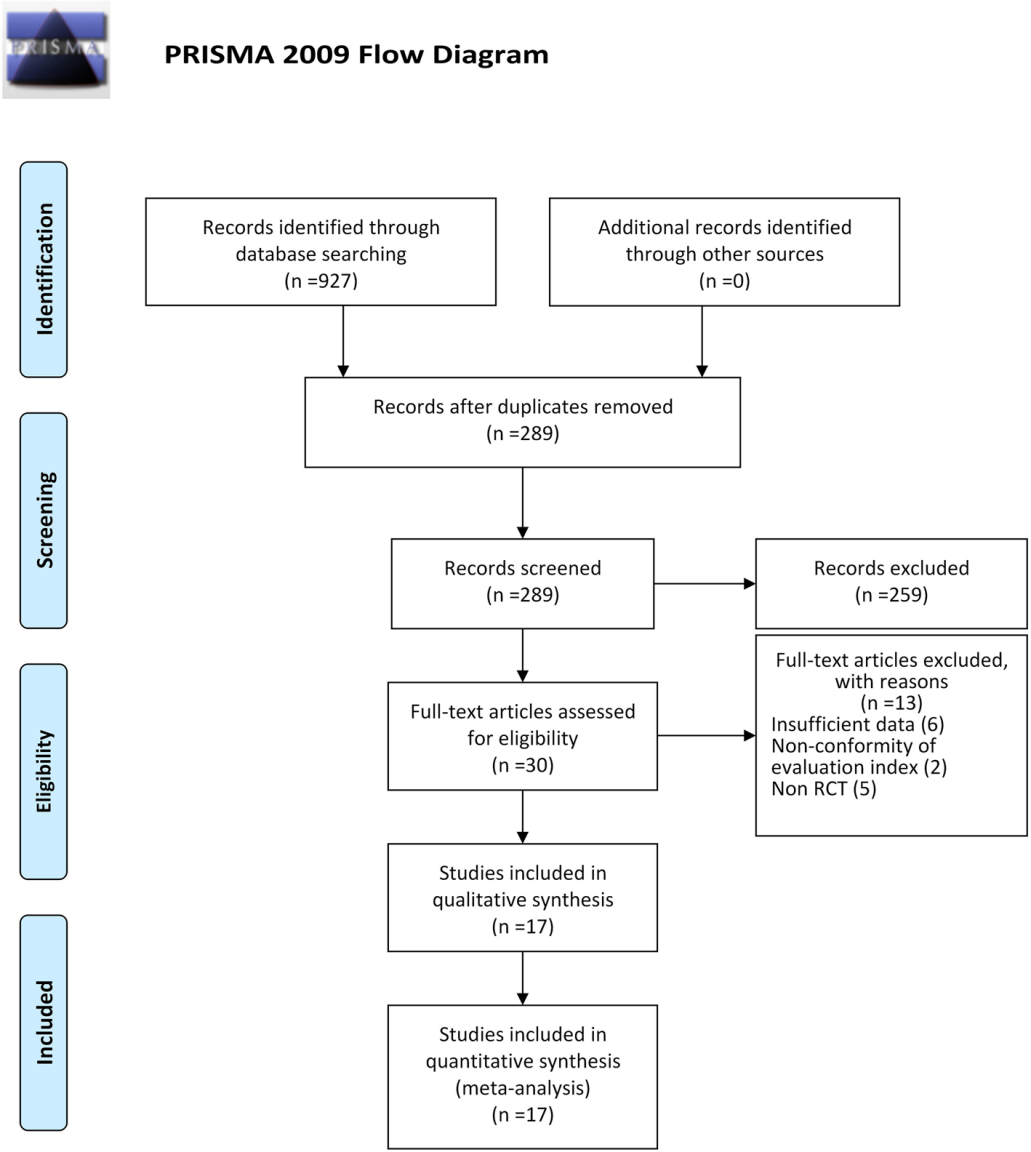
Flow diagram of the study selection process

A total of 927 studies were retrieved by retrieving each data, 638 of which were excluded according to the title and abstract. After removing duplicate results, the full text of 30 articles was checked. Following this, 13 articles were excluded for not meeting our inclusion criteria and 17 articles were eligible for inclusion in the meta-analysis. The publication years of the included studies were from 2006 to 2023, the sample size is between 20—160. The screening process and results of the literature are shown in Fig. 1.

### Study characteristics

A total of 1131 patients with RCT were included in the 17 included studies [20–36]. The research characteristics of each included study, encompassing the sample size in both the experimental and control groups, participant age, disease duration, type and dosage of extracorporeal shock wave therapy, intervention duration, as well as outcome evaluation indicators are presented in Table 1.

**Table 1:** Characters of included studies in the meta-analysis

Author Year (Ref.)	Sample size (T/C)	Age, years (T/C)	Duration of symptoms (T/C)	Type of intervention (T/C)		Types and Brands of ESWT	Dose of EWST	Duration of intervention	Main Outcomes
Shao 2023	19/19	51.4 ± 8.2/55.7 ± 9.2	6.4 ± 3.5/5.2 ± 4.2m	ESWT + Routine rehabilitation training	Routine rehabilitation training	Radial shock wave, Swiss Dolor-Clast, EMS	Each point received 1000 impulses, and a total of 2000 impulses were applied. A total of 0.08mJ/mm^2^ energy flux density was applied, with a pressure of 2.5 bars and a frequency of 6 Hz	**5 w** Shock wave therapy was administered once a week for a total of 5 sessions	VAS, ROM, UCLA, ASES, SNQ
Xi 2022	31/31	69.21 ± 4.83/69.21 ± 4.83	18. 02 ± 8. 64/18. 64 ± 9. 11m	ESWT + Routine rehabilitation training	Routine rehabilitation training	Focused shock wave, Zimmer Company, enPuls Version 2.0, Germany	The probe is 15 mm straight, the frequency was 10 Hz, the energy flow density was 0.08 mJ/mm^2^, and the total number of shocks was 2000 times	**4 w** Shock wave therapy was administered once a week, for a total of 4 weeks	VAS, TER, ROM, UCLA
Fatima 2022 [24]	20/20	48.7 ± 6.74/49.8 ± 7.54	-	ESWT + Routine rehabilitation training	Routine rehabilitation training	Radial SWT BLT-6000 device (UK)	Each ESWT session was given for 15–20 min in which patients have been treated by 2000 shocks with 120 Hz. The procedure could be slightly painful in the first session, so a low-energy density of 0.03mJ/mm^2^ was given for the first five minutes and then progressively increasing to 0.32mJ/mm^2^	**6 w** Shock wave therapy was administered twice a week for six weeks	CMS, VAS, WORC
Xu 2022	34/40	52.69 ± 6.10/53.04 ± 6.33	10.49 ± 2.77/10.26 ± 2.70d	ESWT + Routine rehabilitation training	Routine rehabilitation training	Radial shock wave, Swiss Dolor-Clast, EMS	The probe is 15 mm straight, the frequency is 5 Hz, the impact times of each pain point is 2500 times	**3 w** Shock wave therapy was administered once every 2 days, a total of 10 times	CMS, UCLA, ASES
Zhang 2021	60/60	61.02 ± 1.07/60.53 ± 1.49	-	ESWT + Routine rehabilitation training	Routine rehabilitation training	Focused shock wave	Low energy shock wave, energy range: 0.08–0.28mJ/mm^2^, 1200–1500 times	**4 w** Shock wave therapy was administered once a week, for a total of 4 weeks	VAS, CMS, TER
Luo 2021	80/80	57.1 ± 6.9/56.5 ± 6.4	-	ESWT + Routine rehabilitation training	Routine rehabilitation training	CJB divergent extracorporeal shock wave	The treatment pressure was 3.5 ~ 5.0 Bar, the number of shocks was 2000 ~ 3500, and the frequency was 12 ~ 15 Hz	**5 w** Shock wave therapy was administered once a week, for a total of 5 weeks	VAS, CMS, UCLA
Zhu 2021	20/20	57.60 ± 7.21/57.55 ± 6.48	48.05 ± 4.66/48.3 ± 3.76d	ESWT + Routine rehabilitation training	Routine rehabilitation training	Electromagnetic extracorporeal shock wave (Israeli Medispec series Radialspec model)	Select a 15 mm electrode head, use a low frequency of 5 Hz, energy level 1 (60 mJ), impact 2000 times, 500 times per point, and the rest of the uniform impact	**6 w** Shock wave therapy was administered once a week, for a total of 6 weeks	VAS
Zheng 2020	29/28	36.3 ± 5.6/38.5 ± 5.4	3 weeks to 3 months	ESWT + Routine rehabilitation training	Routine rehabilitation training	Radial shock wave, STORZ of Switzerland	The treatment probe with a diameter of 15 mm was fixed at 8 Hz and the intensity was controlled at 2.0 ~ 3.2 bar. Each treatment had a therapeutic impact of 4000r	**4 w** Twice a week for 4 weeks, a total of 8 times a course of treatment	UCLA, VAS, ROM
Zhao 2020	27/27	43.65 ± 3.27/42.34 ± 3.45	8.5 ± 1.02/8.86 ± 0.93m	ESWT + Floating needle therapy	Floating needle therapy	Focused shock wave	Set the pulse to 2000, set the energy to 0.25 mJ/mm^2^, and set the frequency to 3.0 Hz	**4 w** Twice a week for 4 weeks, a total of 8 times a course of treatment	VAS, ROM, SF-36, TER
Tian 2020	28/28	52.21 ± 6.47/55.07 ± 6.47	30.43 ± 17.91/32.60 ± 16.14d	ESWT + Routine rehabilitation training	Routine rehabilitation training	Radial shock wave, Swiss Dolor-Clast, EMS	The treatment probe with a diameter of 15 mm was fixed at 10 Hz and the intensity was controlled at 1.8 ~ 3.5 bar the energy flow density (0.12 ~ 0.25mJ/mm^2^), and the impact of each treatment is 2000 times	**8 w** Each treatment interval was 1 week, and each patient in the observation group received 4 treatments	VAS, UCLA, TER
Duymaz 2019 [22]	40/40	54.33 ± 9.88/51.31 ± 8.86	> 12m	ESWT + Routine rehabilitation training	Routine rehabilitation training	Radial shock wave, Shock Master 500 device, (GymnaUniphy NV, Bilzen, Belgium)	1500 shocks with a frequency of 150 shocks per minute. Since pain could occur mostly during the first treatment, all patients were treated with a low energy density of 0.03 mJ/mm^2^ for the first five minutes, which was then progressively increased to 0.28 mJ/mm^2^	**4 w** Once a week for four weeks in total	VAS, ROM
Chen 2018	22/16	19.14 ± 3.04/19.63 ± 3.05	2.16 ± 1.55/2.16 ± 1.09y	ESWT + Routine rehabilitation training	Routine rehabilitation training	Radial shock wave, Switzerland STORZ, DUOLITH® SD1 ultra	Pulse 2000, energy 0.25mJ/mm^2^, frequency 3.0Hz	**4 w** Shock wave therapy was administered twice a week, for a total of 4 weeks	VAS, ASES, TER
Su 2018	32/30	56.88 ± 10.45/57.87 ± 12.43	> 3m	ESWT + Routine rehabilitation training	Routine rehabilitation training	Focused shock wave	The parameter is 0.08 mJ/mm^2^, 2000 times, and the frequency is 8 Hz	**4 w** Shock wave therapy was administered once a week, for a total of 4 weeks	VAS, CMS
Xie 2017	30/30	68.7 ± 6.9/67.5 ± 9.3	111.8 ± 69.8/104.6 ± 53.4m	ESWT + Routine rehabilitation training	Routine rehabilitation training	Radial shock wave, Switzerland, STORZ	The treatment frequency was 14 Hz, the pressure was 1.0 ~ 2.5 bar, the energy flux density (EFD) was 0.38 mJ/mm^2^, the treatment probe was 15 mm, and each shock was 2,000 times	**8 w** Shock wave therapy was administered once a week, for a total of 8 weeks	VAS, CMS, TER
Wang 2013	40/40	49.2 ± 10.6/47.6 ± 9.8	12.8 ± 4.2/12.3 ± 3.8w	ESWT + Routine rehabilitation training	Routine rehabilitation training	Radial shock wave, MASTERPULS MP-100 is produced by STORZ, Switzerland	Voltage 15 ~ 25 kV, 2000 shocks per time, power energy density 0.16 mJ/mm^2^	**4 w** Shock wave therapy was administered once a week, for a total of 4 weeks	CMS, VAS, ROM
Galasso 2012 [25]	11/9	50.7 ± 8.44/51.11 ± 13.26	45.36 ± 34.33/61.22 ± 24.04m	ESWT + Routine rehabilitation training	Routine rehabilitation training	ModulithW SLK system (Storz Medical AG, Tagerwilen, Switzerland)	The treatment regimen required administration of two treatment sessions, each consisting of 3000 shockwaves at an energy flux density of 0.068 mJ/mm^2^	**6 w** Shock wave therapy was administered once a week, for a total of 6 weeks	CMS
Cacchio 2006 [20]	45/45	56.12 ± 1.98/56.42 ± 2.09	14 ± 4.95/13 ± 5.03m	ESWT + Routine rehabilitation training	Routine rehabilitation training	Radial shock wave	2500 impulses per session (500 impulses with a pressure of 1.5 bar and a frequency of 4.5 Hz and 2000 impulses with a pressure of 2.5 bar and a frequency of 10 Hz), an EFD of 0.10 mJ/mm^2^, and a fixed impulse time of 2 ms	**4 w** Each subject in the treatment group received 4 sessions at 1-week intervals	UCLA, VAS, SPADI

*T* Trial group; *C* Control group; *ESWT* Extracorporeal shock wave therapy; *VAS* Visual Analogue Scale/Score; *CMS* Constant-Murley score; *UCLA* University of California Los Angeles score; *ROM* Range of motion; *TER* Total effective rate; *ASES* American Shoulder and Elbow Surgeons form; *SPADI* Shoulder Pain & Disability Index; *EFD* Energy flux density; *WORC* Western Ontario Rotator Cuff; *SF-36* 36-item Short-Form; *SNQ* Signal/Noise Quotient

### Risk of bias in studies

All studies were assessed using the ROB 2.0. It was found that five studies were of low-risk bias [20, 22, 23, 25, 29], and twelve studies were conducted as controlled clinical trials, raising concerns about potential bias in several criteria [21, 24, 26–28, 30–36]. The risk of bias assessed by the study are shown in Table 2.

**Table 2:** The risk of bias of RCTs included and evaluated through Rob 2.0

Author, year	Randomization process	Deviation from intended interventions	Missing Outcome data	Measurement of the outcome	Selection of the reported result	Overall
Shao 2023	Low risk	Low risk	Low risk	Low risk	Low risk	Low risk
Xi 2022	Some concerns	Low risk	Low risk	Low risk	Low risk	Some concerns
Fatima 2022 [24]	Low risk	Some concerns	Low risk	Low risk	Low risk	Some concerns
Xu 2022	Some concerns	Some concerns	Low risk	Low risk	Low risk	Some concerns
Zhang 2021	Some concerns	Some concerns	Low risk	Low risk	Low risk	Some concerns
Luo 2021	Some concerns	Some concerns	Low risk	Low risk	Low risk	Some concerns
Zhu 2021	Low risk	Some concerns	Low risk	Low risk	Low risk	Some concerns
Zheng 2020	Low risk	Some concerns	Low risk	Low risk	Low risk	Some concerns
Zhao 2020	Some concerns	Some concerns	Low risk	Low risk	Low risk	Some concerns
Tian 2020	Some concerns	Some concerns	Low risk	Low risk	Low risk	Some concerns
Duymaz 2019 [22]	Low risk	Low risk	Low risk	Low risk	Low risk	Low risk
Chen 2018	Low risk	Low risk	Low risk	Low risk	Low risk	Low risk
Su 2018	Low risk	Some concerns	Low risk	Low risk	Low risk	Some concerns
Xie 2017	Some concerns	Some concerns	Low risk	Low risk	Low risk	Some concerns
Wang 2013	Low risk	Some concerns	Low risk	Low risk	Low risk	Some concerns
Galasso 2012 [25]	Low risk	Low risk	Low risk	Low risk	Low risk	Low risk
Cacchio 2006 [20]	Low risk	Low risk	Low risk	Low risk	Low risk	Low risk

### Results of syntheses

#### VAS

**Fig. 2:**
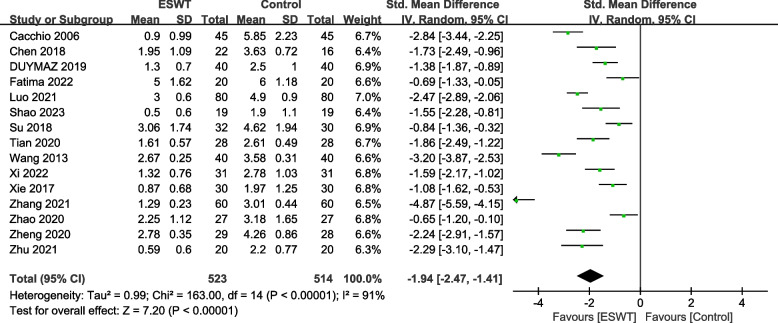
Forest plot of VAS on shoulder pain

Fifteen studies [20–24, 26–29, 31–36] involving 1037 patients used the VAS to assess the pain relief effect. The heterogeneity results showed that there was heterogeneity among the studies (*P* < 0.00001, I^2^ = 91%), and the random effects model was used for meta-analysis. The results showed that there was a significant difference in pain reduction between the ESWT group and the control group (SMD = -1.94, 95% CI -2.47, -1.41, *P* < 0.00001) (Fig. 2).

#### CMS

**Fig. 3:**
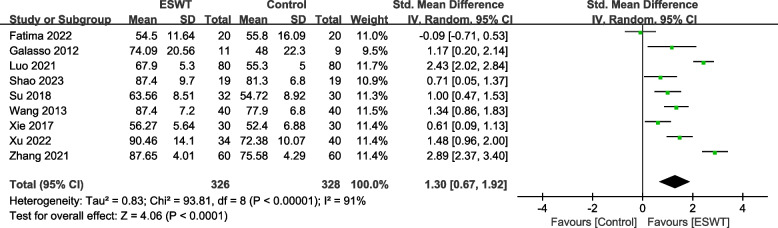
Forest plot of CMS on shoulder function

Nine studies [21, 24–26, 28–30, 32, 35] involving 654 patients used the CMS to assess the effect of shoulder function. The heterogeneity results showed that there was heterogeneity among the studies (*P* < 0.00001, I^2^ = 91%), and the random effects model was used for meta-analysis. The results showed that there was a significant difference in the improvement of shoulder function between the ESWT group and the control group (SMD = 1.30, 95% CI 0.67, 1.92, *P* <0.0001) (Fig. 3).

#### UCLA

**Fig. 4:**
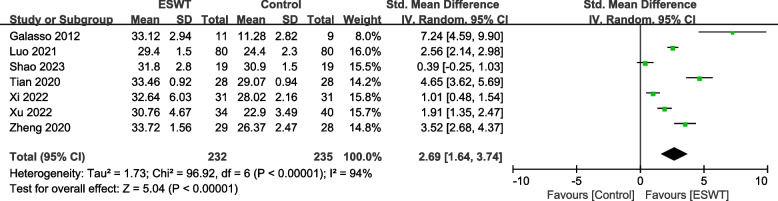
Forest plot of UCLA on shoulder function

Seven studies [25, 27, 29, 30, 33–35] involving 467 patients used the UCLA to assess the effect of shoulder function. The heterogeneity results showed that there was heterogeneity among the studies (*P* < 0.00001, I^2^ = 94%), and the random effects model was used for meta-analysis. The results showed that there was a significant difference in the improvement of shoulder function between the ESWT group and the control group (SMD = 2.69, 95% CI 1.64, 3.74, *P* < 0.00001) (Fig. 4).

#### ROM

**Fig. 5:**
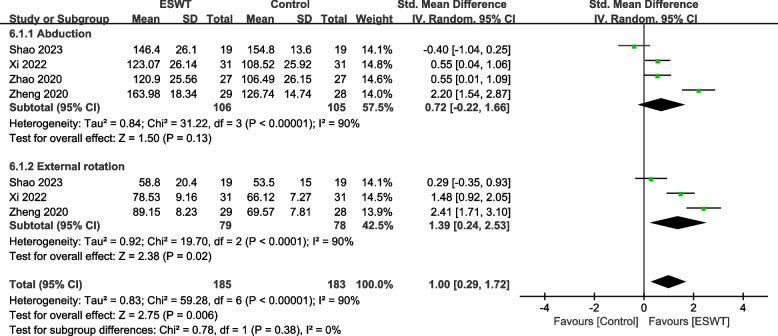
Forest plot of ROM on shoulder function

Four studies [27, 29, 33, 36] involving 211 patients used the ROM-Abduction to assess the Angle of motion of the shoulder joint. The heterogeneity results showed that there was heterogeneity among the studies (*P* < 0.00001, I^2^ = 90%), and the random effects model was used for meta-analysis. The results showed that there was no significant difference in the improvement of shoulder abduction angle between the ESWT group and the control group (SMD = 0.72, 95% CI -0.22, 1.66, *P* = 0.13) (Fig. 5).

Three studies [27, 29, 33] involving 157 patients used the ROM-External rotation to assess the Angle of motion of the shoulder joint. The heterogeneity results showed that there was heterogeneity among the studies (*P* < 0.00001, I^2^ = 90%), and the random effects model was used for meta-analysis. The results showed that there was a significant difference in the improvement of shoulder external rotation angle between the ESWT group and the control group (SMD = 1.00, 95% CI 0.29, 1.72, *P* = 0.02) (Fig. 5).

#### ASES

**Fig. 6:**
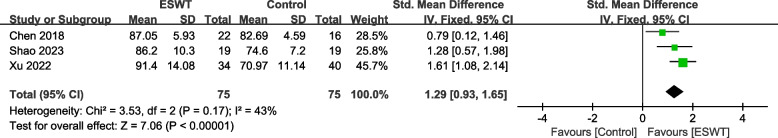
Forest plot of ASES on shoulder function

Three studies [23, 29, 30] involving 150 patients used the ASES to assess the effect of shoulder function. The heterogeneity results showed that there was low heterogeneity among the studies (*P* = 0.17, I^2^ = 43%), and the fixed effects model was used for meta-analysis. The results showed that there was a significant difference in the improvement of shoulder function between the ESWT group and the control group (SMD = 1.29, 95% CI 0.93, 1.65, *P* < 0.00001) (Fig. 6).

#### TER

**Fig. 7:**
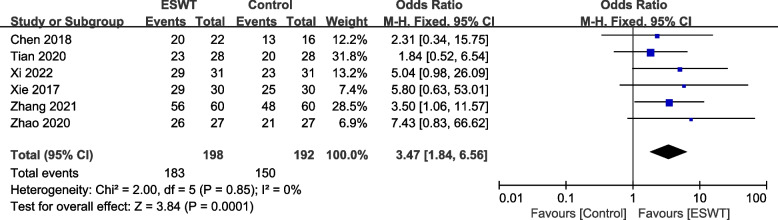
Forest plot of TER on shoulder function

Six studies [23, 28, 32–34, 36] involving 390 subjects reported the TER of RCT recovery. Meta-analysis showed that there was no heterogeneity among the studies (*P* = 0.85, I^2^ = 0%), and there was a significant difference in the effective rate of RCT treatment between the ESWT group and the control group (OR = 3.47, 95% CI: 1.84, 6.56, *P* = 0.0001), indicating that the ESWT group intervention is more effective than the control group (Fig. 7).

### Subgroup analysis

Subgroup analysis was carried out according to the intensity of the shock wave in each study, and those with a shock wave intensity less than or equal to 0.1mj/mm^2^ were divided into one group, and those with an intensity greater than 0.1mj/mm^2^ were divided into a group. Subgroup analysis of VAS and CMS indicators was performed.

### VAS by intensity

**Fig. 8:**
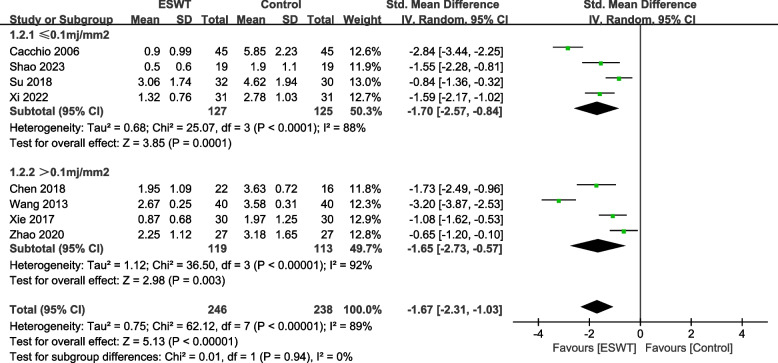
Subgroup by strength–Forest plot of VAS on shoulder pain

Among the VAS indicators, 4 studies [20, 21, 29, 33] involved 252 patients with shock wave intensity less than or equal to 0.1mj/mm^2^, and 4 studies [23, 26, 28, 36] involved 232 patients with shock wave intensity greater than 0.1mj/mm^2^. There was a statistically significant difference between the experimental group and the control group using a shock wave intensity of 0.1mj/mm^2^ or less (SMD = -1.70, 95% CI -2.57, -0.84, *P* = 0.0001, I^2^ = 88%). The difference between the experimental group and the control group using shock wave intensity greater than 0.1mj/mm^2^ was statistically significant (SMD = -1.65, 95% CI -2.73, -0.57, *P* = 0.003, I^2^ = 92%) (Fig. 8).

### CMS by intensity

**Fig. 9:**
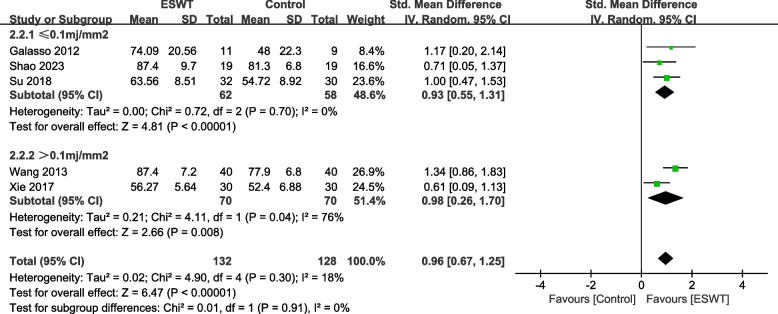
Subgroup by strength–Forest plot of CMS on shoulder function

Among the CMS indicators, 3 studies [21, 25, 29] involved 120 patients with shock wave intensity less than or equal to 0.1mj/mm^2^, and 2 studies [26, 28] involved 140 patients with shock wave intensity greater than 0.1mj/mm^2^. There was a statistically significant difference between the experimental group and the control group using a shock wave intensity of 0.1mj/mm^2^ or less (SMD = 0.93, 95% CI 0.55, 1.31, *P* < 0.00001, I^2^ = 0%). The difference between the experimental group and the control group using shock wave intensity greater than 0.1mj/mm^2^ was statistically significant (SMD = 0.98, 95% CI 0.26, 1.70, *P* = 0.008, I^2^ = 76%) (Fig. 9).

According to each type of shock wave, which can be divided into radial-ESWT (r-ESWT) and focused-ESWT (f-ESWT). Subgroup analysis of VAS and CMS indicatiors was performed.

### VAS by type

**Fig. 10:**
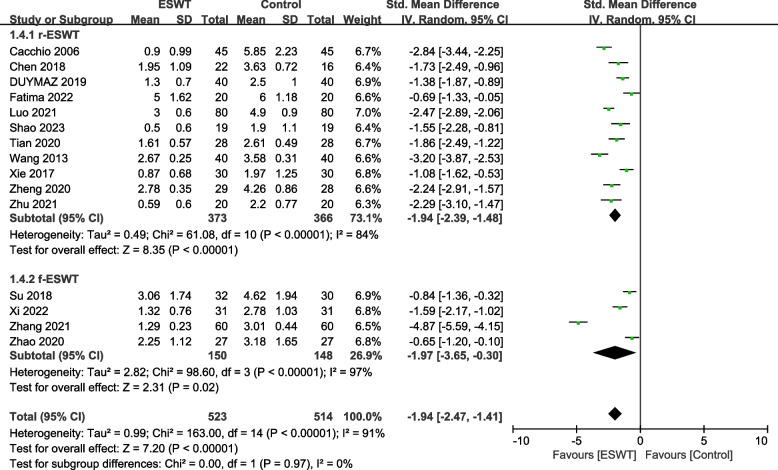
Subgroup by type–Forest plot of VAS on shoulder pain

Among the VAS indicators, 11 studies [20, 22–24, 26–29, 31, 34, 35] involved 739 patients with r-ESWT, and 4 studies [21, 32, 33, 36] involved 298 patients with f-ESWT. There was a statistically significant difference between the experimental group and the control group using r-ESWT (SMD = -1.94, 95% CI -2.39, -1.48, *P* < 0.00001, I^2^ = 84%). The difference between the experimental group and the control group using f-EAWT was statistically significant (SMD = -1.97, 95% CI -3.65, -0.30, *P* = 0.02, I^2^ = 97%) (Fig. 10).

### CMS by type

**Fig. 11:**
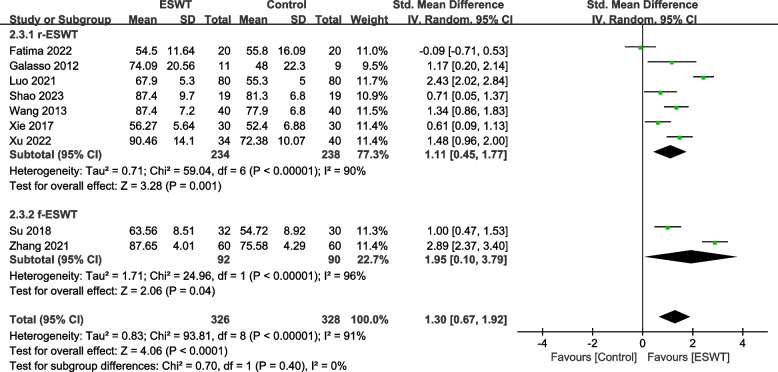
Subgroup by type–Forest plot of CMS on shoulder function

Among the CMS indicators, 7 studies [24–26, 28–30, 35] involved 472 patients with r-ESWT, and 2 studies [21, 32] involved 182 patients with f-ESWT. There was a statistically significant difference between the experimental group and the control group using r-ESWT (SMD = 1.11, 95% CI 0.45, 1.77, *P* = 0.001, I^2^ = 90%). The difference between the experimental group and the control group using f-ESWT was statistically significant (SMD = 1.95, 95% CI 0.10, 3.79, *P* = 0.04, I^2^ = 96%) (Fig. 11).

### Reporting biases

**Fig. 12:**
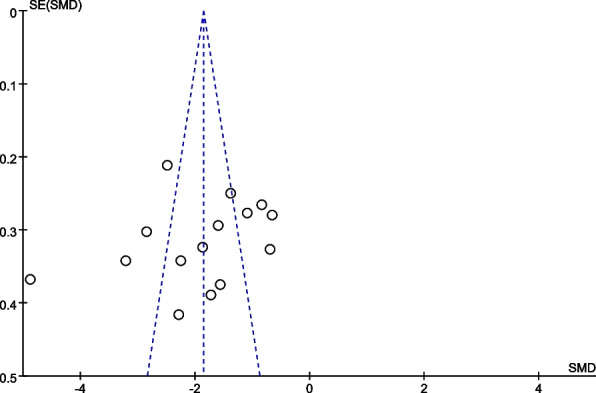
Funnel plot for comparison of VAS between the two groups

Funnel plot was drawn for the studies on VAS with more outcome indicators in the included studies. Most of the VAS studies were distributed within the 95% CI range of the inverted funnel plot. The results show that the distribution is vertically symmetrical, indicating that the publication bias is small (Fig. 12).

## Discussion

RCT is one of the most common musculoskeletal degenerative diseases of aging. The etiology is complex and diverse, with varying clinical treatment approaches. The best treatment method is still uncertain. Surgical treatment has the possibility of secondary infection, and conservative drug treatment is also prone to various adverse reactions, especially for the elderly, who have a higher risk of drug use [37]. Therefore, it is particularly important to choose a safer and more effective treatment method for elderly patients with RCT. Extracorporeal shock waves transmit sound waves to the affected area through the skin. As a non-invasive treatment, it has been gradually used in the treatment of RCT in recent years. The main mechanism of action includes: extracorporeal shock wave can directly use the mechanical effect generated between the local mechanical vibration effect and cavitation to cause changes in human tissues and cells, stimulate blood vessel expansion, and promote regeneration of tendon and soft tissue [38]. inhibit the high-frequency pulse emitted by pain receptors and the transmission of pain signals, improve the water and electrolyte circulation and the metabolism of the treatment area, evacuate local inflammation, and then reduce the load and relieve pain, improve the function of the shoulder joint and increase the ROM of the shoulder joint [39].

To the best of our knowledge, this study represents the first meta-analysis focusing on the impact of ESWT on pain and function among patients with RCT. Our findings indicate that the noninvasive nature of ESWT renders it an efficacious treatment modality for alleviating pain and enhancing function following RCT. These results offer the most robust current evidence regarding the utilization of ESWT in RCT, drawing from available randomized controlled trials. Specifically, our analysis reveals that ESWT significantly reduces shoulder pain and enhances function post-RCT. However, the improvement in shoulder abduction ROM does not exhibit statistically significant differences compared to the control group. Subgroup analyses further demonstrate that ESWT remains effective in mitigating shoulder pain and enhancing shoulder function, irrespective of the administered energy dose.

The goals of RCT treatment are pain control and maintenance of function. Once pain is under control, the function can be maintained with exercises to increase ROM and strengthen the rotator cuff. This meta-analysis demonstrated the superiority of ESWT in terms of clinical pain relief and recovery of shoulder function. Results regarding ESWT on RCT were similar to a previous study by Fatima et al., in which pain was reduced and the effect was maintained for 12 weeks. Although the mechanism by which ESWT improves pain effects is unclear, it has been suggested that ESWT produces oscillations in tissues that improve microcirculation and metabolic activity [40]. The immediate pain reduction after ESWT can be explained by the results of overstimulation analgesia [41]. Furthermore, gender may also influence the effectiveness of ESWT in pain relief. In a retrospective study examining the subjects influenced by RCT, it was observed that among individuals undergoing ESWT alone, males reported higher benefits in pain relief compared to females [42].

Various etiologies of RCT including rotator cuff tendonitis, partial rotator cuff tears, adhesive capsulitis, subscapular bursitis, and complex regional pain syndrome are thought to lead to antifibrotic, anti-inflammatory, and pain-modulating effects [43]. Since RCT includes an inflammatory response, ESWT can eliminate inflammatory factors in the patient's body, relieve pain, promote the early recovery of shoulder joint function, and improve the curative effect. Ko et al. employed a single session of high-energy extracorporeal shockwave therapy (ESWT) with long-term follow-up and demonstrated its efficacy in improving the functional outcome of rotator cuff lesions accompanied by shoulder stiffness. These findings suggest that ESWT represents a simple, effective, and non-invasive treatment option for such a condition [44]. Similar results were also observed in other studies, with significant improvement in pain reduction and shoulder function in the ESWT group compared with the sham group [45, 46]. In addition, the adverse effects of ESWT were dose-dependent and usually limited to temporary increases in pain and local reactions, such as swelling, erythema, petechiae, or small hematomas, and no serious adverse events were reported [47].

Extracorporeal shock waves can also effectively loosen adhesion tissue and relieve soft tissue spasms, thereby increasing the ROM of the shoulder joint. The results of the Meta-analysis showed that the ROM of external rotation of the two groups was significantly improved compared with that before treatment, and the experimental group was significantly better than that of the control group, but the difference in the ROM of abduction was not statistically significant. Firstly, consider that this may be related to the fact that extracorporeal shock waves can effectively improve pain, thereby improving the patient’s exercise time and effect. Secondly, it may also be because extracorporeal shock waves can damage local tissues, promote the production and accumulation of repair factors, and accelerate the vascularization of rotator cuff ischemia. regeneration, thereby speeding up the repair process and improving the stability of the shoulder joint [48, 49]. In short, extracorporeal shock waves can not only effectively improve the pain of RCT, but also improve the ROM of joints more effectively. Admittedly, the improvement in some symptoms in the control group may have been the expected result of the natural healing process.

In a meta-analysis by Steuri et al., ESWT was found to be more effective than sham ESWT in improving function, pain, and active ROM. Studies have shown that ESWT at doses equal to or greater than 0.28 mJ/mm^2^ is more effective in improving shoulder function and reducing pain [50]. In another related study, the experimental group received ESWT in addition to conventional PT intervention, while the control group only underwent conventional PT intervention. Patients receiving ESWT treatment demonstrated a significant improvement in shoulder function compared to the control group [22]. A study comparing ESWT with a placebo treatment also showed a statistically significant improvement in outcomes for the ESWT group [51]. In this study, the control group also showed pain improvement after the intervention, but the pain improvement was negligible compared with the ESWT group, where the difference was statistically significant.

However, in a study comparing placebo ESWT with ESWT in patients with subacromial pain syndrome with supervised exercise, there were no significant differences in primary or secondary outcomes (VAS, CMS) between the two treatment groups. These results suggest that ESWT has no additional effect on supervised exercise in this patient group in the short, medium, or long term. Analyzing causes with negative outcome expectations, frequent use of pain medication, not working from baseline, marital status (single), low self-reported general health, and participation in infrequent supervised exercise classes all predicted poor SPADI results after one year [52]. In a study comparing placebo ESWT and ESWT to RCT, Kolk et al. found that VAS, CMS, and SST scores improved significantly in both groups at 3 and 6 months after treatment, compared with placebo at low doses ESWT does not appear to be effective in reducing symptoms in patients with chronic rotator cuff tendinitis. Therefore, a beneficial effect of ESWT in patients with shoulder tendonitis could not be demonstrated [53]. These results support a previous study by Schmitt et al. ESWT did not improve CMS, SPADI, or pain in patients with noncalcified cuff tendonitis [54].

There may be several explanations for these inconsistent results, such as the high number of variables in the ESWT application (frequency, pressure, treatment interval, etc.), the large heterogeneity of the reported treatment regimens, and the large variation in shock wave intensity. The reliability of blinding in each study is questionable, and although ESWT has been extensively studied, the exact mechanism by which ESWT reduces tendon-related pain is unknown. Theoretical benefits are promoting tissue healing and breaking down calcifications. The intensity of ESWT is measured by energy flux density (EFD), which is generally divided into low-energy, intermediate-energy, and high-energy shockwave therapy, and may also affect the outcome of the treatment. Currently, there is no consensus on the exact dividing point between low-energy and high-energy shockwaves. In general, an EFD of less than 0.08 mJ/mm^2^ corresponds to low energy, while an EFD of a high-energy extracorporeal shock wave is greater than 0.28 mJ/mm^2^. Although the dose–response relationship between low-energy and high-energy ESWT has not been established, studies have shown that high-energy ESWT (> 0.28 mJ/mm^2^) is more likely than low-energy ESWT (< 0.08 mJ/mm^2^) to improve shoulder joints in patients with chronic calcific tendinopathy function and pain relief [55]. The advantage of high-energy ESWT is that it is widely applicable in out-of-hospital settings and is relatively inexpensive. The clinical effect is good, and the treatment has no serious side effects and long-term complications. Generally, however, patients require multiple ESWT treatments to achieve these results. Therefore, further research is needed to better understand the relative efficacy of these treatments.

Previous research findings indicate that both f-ESWT and r-ESWT are superior to placebo in alleviating pain and improving knee joint function [56]. In this study, we conducted a subgroup analysis based on the type of shock wave, revealing that both rESWT and fESWT groups exhibited superior improvements in VAS and CMS compared to the control group. However, Raffaello et al. found, in their investigation of the safety and efficacy of fESWT and rESWT in Lateral elbow tendinopathy (LET) symptoms and wrist extensor strength, that both fESWT and rESWT could potentially improve LET symptoms. ESWT appears to be an effective alternative to conventional therapeutic modalities for treating pain, disability, and muscle injuries associated with LET. Nonetheless, rESWT seems to be less effective and requires more time for pain relief and functional recovery [42, 57]. Therefore, further research is needed to compare the effects of rESWT and fESWT specifically in the context of RCT.

## Prospects

Since extracorporeal shock wave is still a relatively new treatment method, many aspects of ESWT for RCT still need further clinical research and improvement, including the determination of the dose of shock therapy and the formulation of a unified treatment prescription. Therefore, further research and clinical trials may be required to elucidate the ideal parameters conducive to ESWT. The efficacy and safety of ESWT for RCT still need further research and clinical trials to confirm. Accumulating more scientific evidence will help clarify its strengths and limitations in specific cases. The treatment of RCT is an individualized process, and treatment plans need to be formulated according to the specific conditions of patients. Future research will pay more attention to individualized treatment methods. Doctors should consider the patient's condition, symptom severity, physical condition and other factors, and choose the most appropriate treatment based on the latest clinical guidelines and research results. Additionally, ESWT may be combined with other treatments, such as physical therapy, medication, or surgery, for better results. In addition to treatment, future research may pay more attention to the prevention and rehabilitation of RCT. It is possible to reduce the incidence of RCT through preventive measures such as strengthening exercise, improving posture, and avoiding overuse. At the same time, the rehabilitation program for RCT will also be further optimized to improve the effect of rehabilitation and prevent recurrence.

## Limitations

There are certain limitations in this study: (1) There are differences in the brand, and intensity (For example, the energy of rESWT is relatively weaker, with a broader range of wave propagation. The energy of fESWT is stronger but concentrated within a smaller area, enabling deeper penetration into tissues) and the dose of extracorporeal shock waves used in each study, may affect the accuracy of the results. (2) Some studies did not use correct random allocation and concealment methods, which may cause selection bias. (3) Due to language limitations, we only included Chinese and English literature. Therefore, to obtain conclusive evidence, we need to expand the sample and include studies in more languages.

## Conclusions

In summary, the current evidence supports the effectiveness of ESWT for the clinical efficacy of shoulder pain and functional recovery in patients with RCT. ESWT provided better pain relief, functional recovery, and maintenance compared with controls. ESWT may be a promising approach for the treatment of RCT. Due to the limited quality and number of included trials, additional high-quality prospective clinical studies are needed to verify these conclusions.

### Supplementary Information

**Supplementary material 1.**

**Supplementary material 2.**

## Data Availability

All data analysed during this study are included in this published article.
