# Correction: Effect of extracorporeal shockwave therapy for rotator cuff tendinopathy: a systematic review and meta‑analysis

**DOI:** 10.1186/s12891-024-07611-x

**Published:** 2024-07-01

**Authors:** Xiali Xue, Qingfa Song, Xinwei Yang, Amila Kuati, Hao Fu, Yulei Liu, Guoqing Cui

**Affiliations:** 1 School of Sports Medicine and Health, Chengdu Sport University, Chengdu, China; 2 Department of Sports Medicine, Peking University Third Hospital, Institute of Sports Medicine of Peking University, Beijing, China; 3 Chengdu University of Traditional Chinese Medicine, Chengdu, China; 4 Department of Rehabilitation, Peking University Third Hospital, Beijing, 100191 China

**Correction:** ***BMC Musculoskelet Disord*** **25, 357 (2024)**

10.1186/s12891-024-07445-7

Following publication of the original article [1], the authors reported that the 6th author was omitted from the author group. Yulei Liu (one of the corresponding authors) has been added to the author group and is presented correctly in this correction article.

Yulei Liu’s affiliation: *Department of Sports Medicine, Peking University Third Hospital, Institute of Sports Medicine of Peking University, Beijing, China*.

The original article [1] has been corrected.
