# Supplementary material for: Effect of extracorporeal shockwave therapy for rotator cuff tendinopathy: a systematic review and meta-analysis
Source: BMC Musculoskelet Disord. 2024 May 4;25:357. doi: 10.1186/s12891-024-07445-7 (PMC11069249; doi:10.1186/s12891-024-07445-7)
Supplement: Supplementary file 2 — Supplementary material 2. [file 12891_2024_7445_MOESM2_ESM.docx]

**Supplementary Material 2. PubMed search strategy.**

| **# No** | **Searches** |
| --- | --- |
| **#1** | “Rotator Cuff Injuries”[Mesh] |
| **#2** | “Cuff Injury, Rotator”[Title/Abstract] OR “Injury, Rotator Cuff”[Title/Abstract] OR “Rotator Cuff Injury”[Title/Abstract] OR “Rotator Cuff Tears”[Title/Abstract] OR “Rotator Cuff Tear”[Title/Abstract] OR “Tear, Rotator Cuff”[Title/Abstract] OR “Tears, Rotator Cuff”[Title/Abstract] OR “Rotator Cuff Tendinosis”[Title/Abstract] OR “Rotator Cuff Tendinoses”[Title/Abstract] OR “Tendinoses, Rotator Cuff”[Title/Abstract] OR “Tendinosis, Rotator Cuff”[Title/Abstract] OR “Rotator Cuff Tendinitis”[Title/Abstract] OR “Rotator Cuff Tendinitides”[Title/Abstract] OR “Tendinitis, Rotator Cuff”[Title/Abstract] |
| **#3** | **#**1 OR **#**2 |
| **#4** | “Extracorporeal Shockwave Therapy”[Mesh] |
| **#5** | “Extracorporeal Shockwave Therapies”[Title/Abstract] OR “Shockwave Therapies, Extracorporeal”[Title/Abstract] OR “Shockwave Therapy, Extracorporeal”[Title/Abstract] OR “Therapy, Extracorporeal Shockwave”[Title/Abstract] OR “Shock Wave Therapy”[Title/Abstract] OR “Shock Wave Therapies”[Title/Abstract] OR “Therapy, Shock Wave”[Title/Abstract] OR “Extracorporeal Shock Wave Therapy”[Title/Abstract] OR “Extracorporeal High-Intensity Focused Ultrasound Therapy”[Title/Abstract] OR “Extracorporeal High Intensity Focused Ultrasound Therapy”[Title/Abstract] OR “HIFU Therapy”[Title/Abstract] OR “HIFU Therapies”[Title/Abstract] OR “Therapy, HIFU”[Title/Abstract] OR “High-Intensity Focused Ultrasound Therapy”[Title/Abstract] OR “High Intensity Focused Ultrasound Therapy”[Title/Abstract] |
| **#6** | **#**4 OR **#**5 |
| **#7** | “Randomized Controlled Trial”[Mesh] |
| **#8** | “Randomized Controlled Trial”[Title/Abstract] OR “Controlled Clinical Trials, Randomized”[Title/Abstract] OR “Clinical Trials, Randomized”[Title/Abstract] OR “Trials, Randomized Clinical”[Title/Abstract] OR “Clinical trial”[Title/Abstract] OR “Clinical trials”[Title/Abstract] |
| **#9** | **#**7 OR **#**8 |
| **#10** | **#**3 AND **#**6 AND **#**9 |
